# Veterinary clients value animal welfare and environmental sustainability in pet food choices

**DOI:** 10.3389/fvets.2026.1735331

**Published:** 2026-02-10

**Authors:** Madison Clark, Danielle Scott, Caroline Kern-Allely, Aurora Koren, Kapahi Kawai Puaa, Jeremy Delcambre, Colleen Duncan

**Affiliations:** 1 College of Veterinary Medicine and Biological Sciences, Colorado State University, Fort Collins, CO, United States; 2 Colorado School of Public Health, Fort Collins, CO, United States; 3 School of Veterinary Medicine, Louisiana State University, Baton Rouge, LA, United States

**Keywords:** animal welfare, environmental sustainability, pet food, veterinary, veterinary team

## Abstract

Pet food, due to its general abundance and use of animal protein, makes a substantial contribution to the environmental ‘pawprint’ of dog and cat ownership. As pet owners are increasingly interested in sustainability issues, which include both the physical environment and animal welfare, our objective was to identify how veterinary teams can support pet owners to make sustainable feeding choices. We surveyed 1,066 people on perceptions of these topics and the role their veterinary team could fill. While people value both factors when choosing pet food, animal welfare was significantly more important in driving pet owners’ choices relative to the environment. We found that pet owners consider the veterinary team, particularly veterinarians, to be a trusted source of information for animal welfare and environmental sustainability. Finally, third-party certifications are well received by both familiar and introduced individuals and could be a powerful tool in these conversations. This study highlights opportunities for veterinary clinics to advance sustainability efforts in the animal health sector.

## 1 Introduction

The health of people, animals, and the environment are inextricably linked, a principle captured in the One Health framework. Today, environmental degradation, through processes such as climate change (1), biodiversity loss (2), and pollution (3), undermines the ecological systems that sustain life and directly impacts both human and animal well-being. Efforts to protect and restore the environment therefore generate broad benefits across species and communities.

The ways we care for companion animals have meaningful impacts on environmental health. Pet food, for example, contributes 56–151 million Tonnes (Mt) CO_2_ equivalent (eq) annually (4), which is roughly comparable to powering 7.5–20 million homes for 1 year. In the US, where many households own pets, food consumption by dogs and cats accounts for 25–30% of the environmental impacts from the animal production industry, in terms of the use of land, water, fossil fuel, phosphate, and biocides (5). Importantly, many of the same supply chains that drive environmental impacts in pet food production also raise animal welfare (AW) concerns. Surveyed pet owners report significantly greater concern for the welfare of animals in intensive production systems than non-pet owners (6) and demonstrate a willingness to pay more for animal welfare labeled products (7). As the environmental impacts of pet food largely depends on the type and composition of these foods (8, 9) choices that owners make regarding what to feed their pets can be very impactful.

Pet food choice involves complex decision-making processes influenced by nutritional, ethical, and environmental considerations. Concern for production animal welfare (10, 11) and environmental sustainability (ES) (12, 13) has grown, particularly among younger pet owners, prompting the pet food industry to respond with more options (14). As trusted advisors, veterinarians are well-positioned to guide pet owners toward more sustainable and welfare-conscious feeding choices (15, 16). However, communication strategies must align with diverse client values and a range of client backgrounds.

Recently, a large-scale survey in Germany found that appeals focused on AW were more effective than those centered on ES in garnering public support for policy interventions related to meat consumption (17). While AW framing appears effective in prompting sustainability considerations in human food, its impact on pet food decisions is less understood, particularly given the emotional nature of the human–animal bond and the influence of veterinary advice. Our study aimed to identify how veterinary teams can support pet owners in making sustainable feeding choices for their pets. Our goal is to inform veterinary communication strategies that effectively integrate AW and ES framing to promote sustainability in ways that align with client values and the profession’s broader sustainability goals (18).

## 2 Materials and methods

We distributed an online, anonymous, 21-question survey (Supplementary material 1) to US pet owners who utilize veterinary services regarding: (1) the general *importance* of ES and AW in pet food decisions; (2) *barriers* to purchasing pet food; (3) the role of *third-party certifications*; and (4) *trust* in veterinary staff as messengers. The survey was distributed through Centiment^1^ to a panel of 1,066 U. S. pet owners, 18 years and older, who take their dog and/or cat to the veterinarian. The panel was balanced (+/− 5% of U. S. Census) for age, gender, and region. The survey was comprised of Likert scale, yes/no, and select-all-that-apply questions. All survey questions required a response with the exception of skip logic questions. Descriptive and comparative analysis (chi-squared tests, ordinal logistic regression, and rho Spearmans) was done using R Version 4.5.1 (19). The survey protocol was reviewed by Colorado State University’s Institutional Review Board and deemed exempt from full review.

## 3 Results

We received a total of 1,066 responses. Respondents were distributed across all age brackets, income levels, education levels, and community types (Table 1). All respondents reported having a small animal who received veterinary care and are included in the final analysis. Of these, 20.4% were cat owners only (217/1066); 43.9% were dog owners only (468/1066), and 35.7% owned both (381/1066).

**Table 1:** Responses to demographic questions regarding age, education, income, community, and pet ownership.

**Category**	**Count (Percent %)**
**Total**	1066 (100.0%)
**Age**	
18-24	126 (11.8%)
25-34	198 (18.6%)
35-44	189 (17.7%)
45-54	181 (17.0%)
55-64	189 (17.7%)
65 or older	183 (17.2%)
**Education**	
Some high school	32 (3.0%)
High school graduate	340 (31.9%)
Two-year associate’s degree	100 (9.4%)
Some college	243 (22.8%)
Four-year bachelor’s degree	216 (20.3%)
Graduate or professional degree	134 (12.6%)
Prefer not to answer	1 (0.1%)
**Income**	
Less than $20,000	156 (14.6%)
$20,000 to $34,999	193 (18.1%)
$35,000 to $49,999	146 (13.7%)
$50,000 to $74,999	229 (21.5%)
$75,000 to $99,999	113 (10.6%)
$100,000 to $149,999	128 (12.0%)
$150,000 to $199,999	43 (4.0%)
$200,000 or more	40 (3.8%)
Prefer not to answer	18 (1.7%)
**Community**	
Rural	237 (22.2%)
Suburban	474 (44.5%)
Urban	355 (33.3%)
**Pet**	
Cat(s)	217 (20.4%)
Dog(s)	468 (43.9%)
Cat(s) and dog(s)	381 (35.7%)

**Figure 1:**
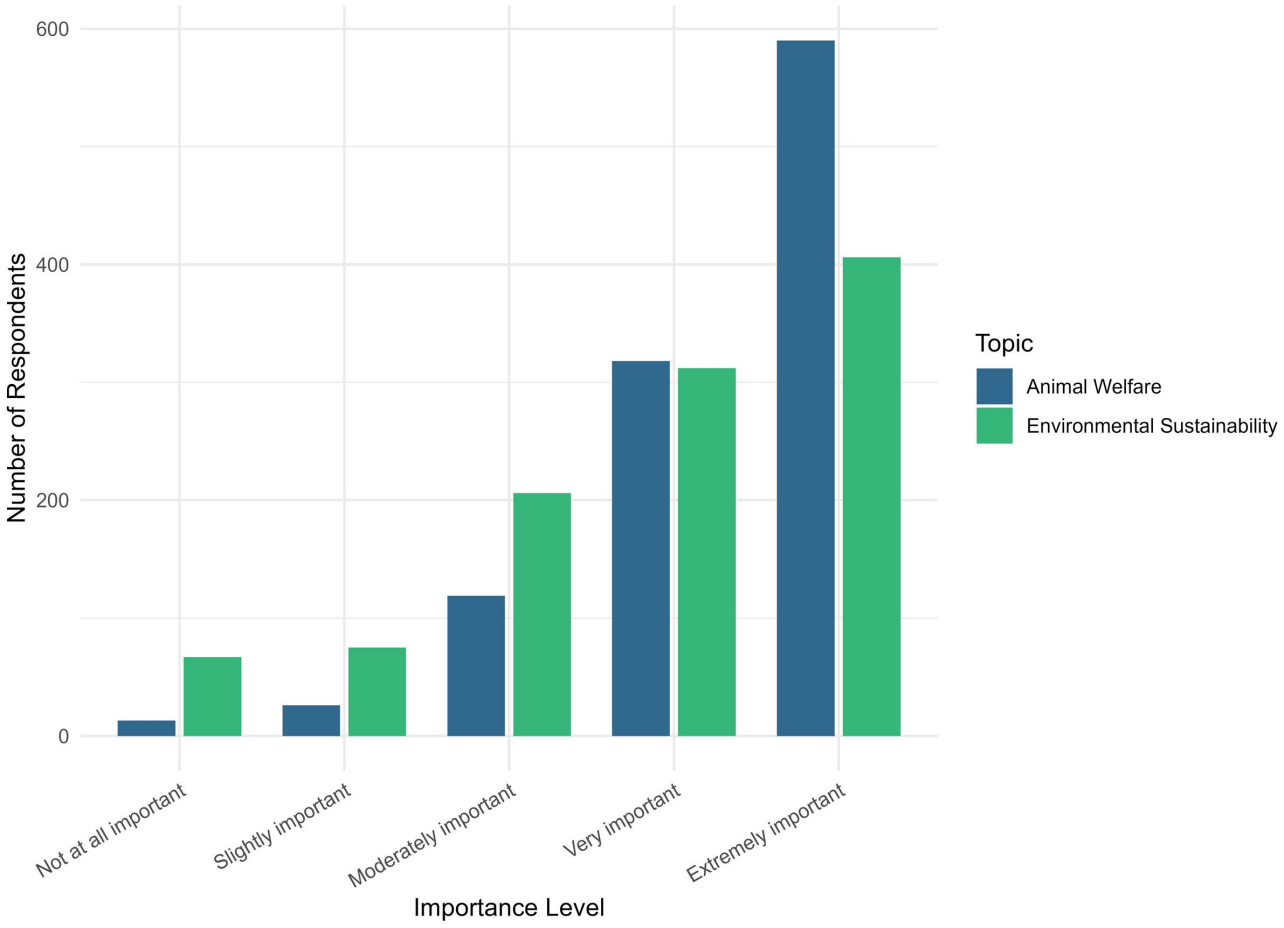
Frequency of respondent choices regarding their perceived importance of environmental sustainability and animal welfare when making pet food decisions.

**Figure 2:**
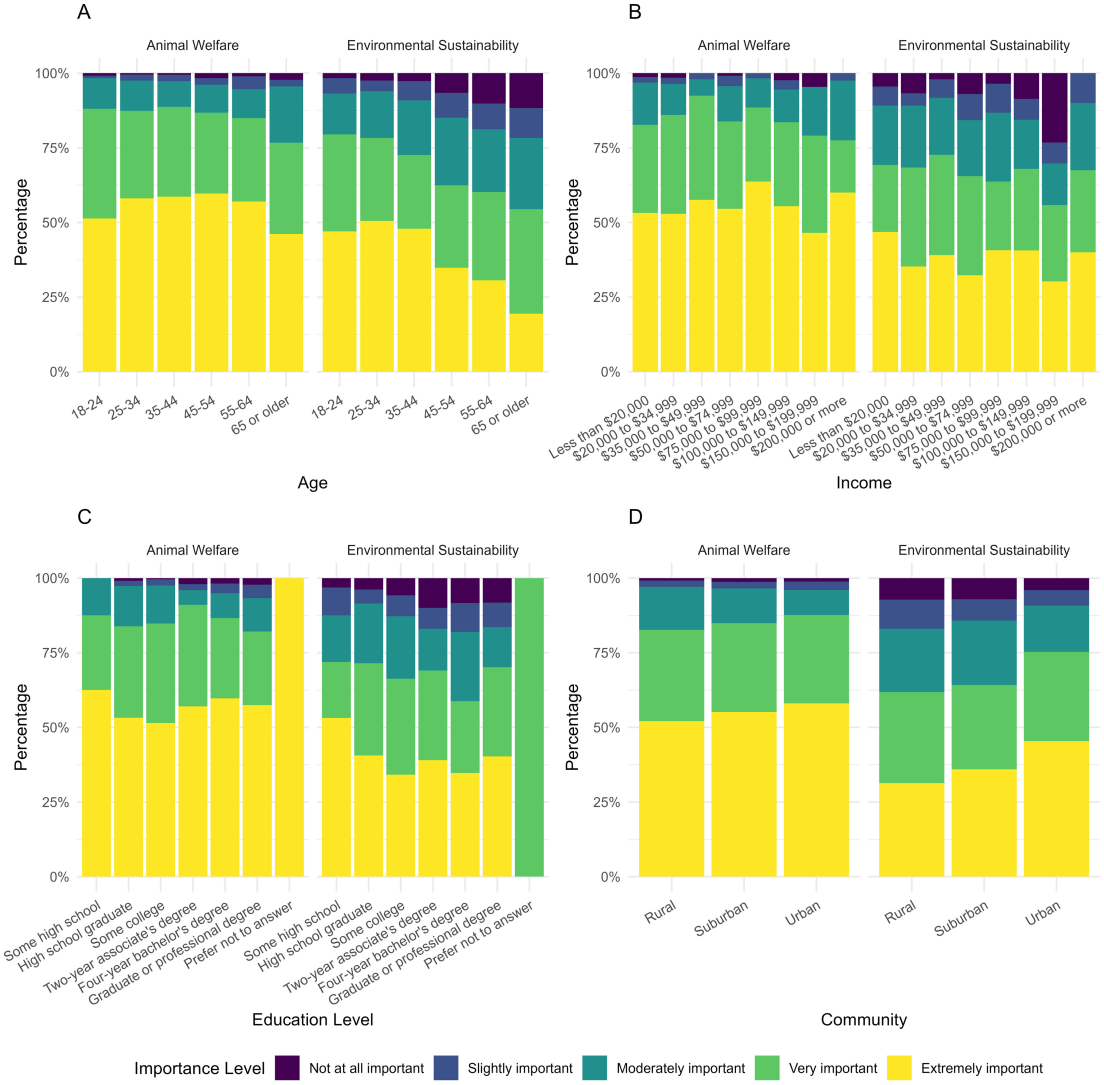
Frequency of response regarding importance of animal welfare and environmental sustainability categorized by **(A)** age, **(B)** income, **(C)** education, and **(D)** community.

We asked participants about the importance of ES and AW when making pet food decisions (Figure 1). Both ES and AW were viewed as very or extremely important by most individuals (ES = 70.1%; AW = 81.1%). AW was significantly more important than ES in driving purchases (OLR, v = 0.439881, *p <* 0.001). We also compared the importance of ES and AW by demographic groups (Figure 2). ES was variably influenced by demographics. Increased education (ρs, correlation = −0.0665, *p <* 0.05) and age (ρs, correlation = −0.246, *p <* 0.001) showed a negative correlation with importance for ES. Those from urban areas rated ES significantly higher than suburban (OLR, v = 0.4148637, *p <* 0.05) or rural areas (OLR, v = 0.5858419, *p <* 0.001). Income was not significantly associated with the importance of either topic. No demographic significantly influenced AW.

**Figure 3:**
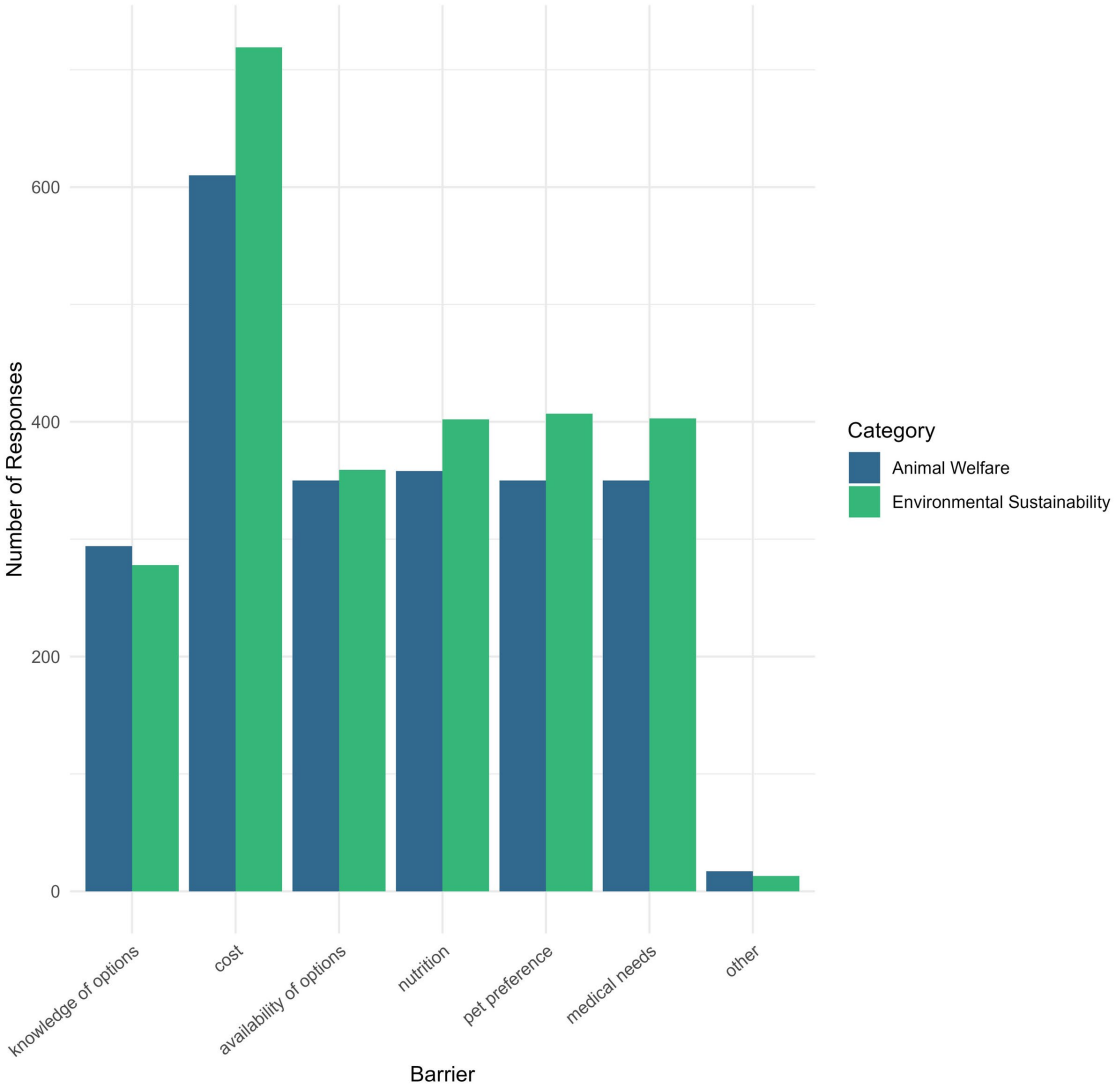
Frequency of respondent choice regarding barriers to purchasing pet food for environmental sustainability (ES) or animal welfare (AW) reasons (multiple select).

Regarding barriers to purchase pet food within either the ES or AW category (Figure 3), participants chose cost (*p <* 0.001), pet preference (*p <* 0.05), and medical needs (*p <* 0.05) significantly more for ES than AW. There were significant differences between the barriers for both ES (X-squared, 265.62, df = 5, *p <* 0.001) and AW (X-squared, 164.3, df = 5, *p <* 0.001), with the majority of participants choosing cost as a barrier to purchase (ES = 67.4%, AW = 57.2%).

Most participants were unaware of third-party certifications for AW (61.9%, 660/1066) or ES (61.7%, 658/1066). Of those who were aware of such certifications, the majority strongly or somewhat agreed that they impacted purchasing decisions (62.7%, 292/466). Of those who were not aware of them, a majority indicated they are now somewhat likely or very likely to utilize third-party certifications in pet food decisions (64.7%, 388/600).

**Figure 4:**
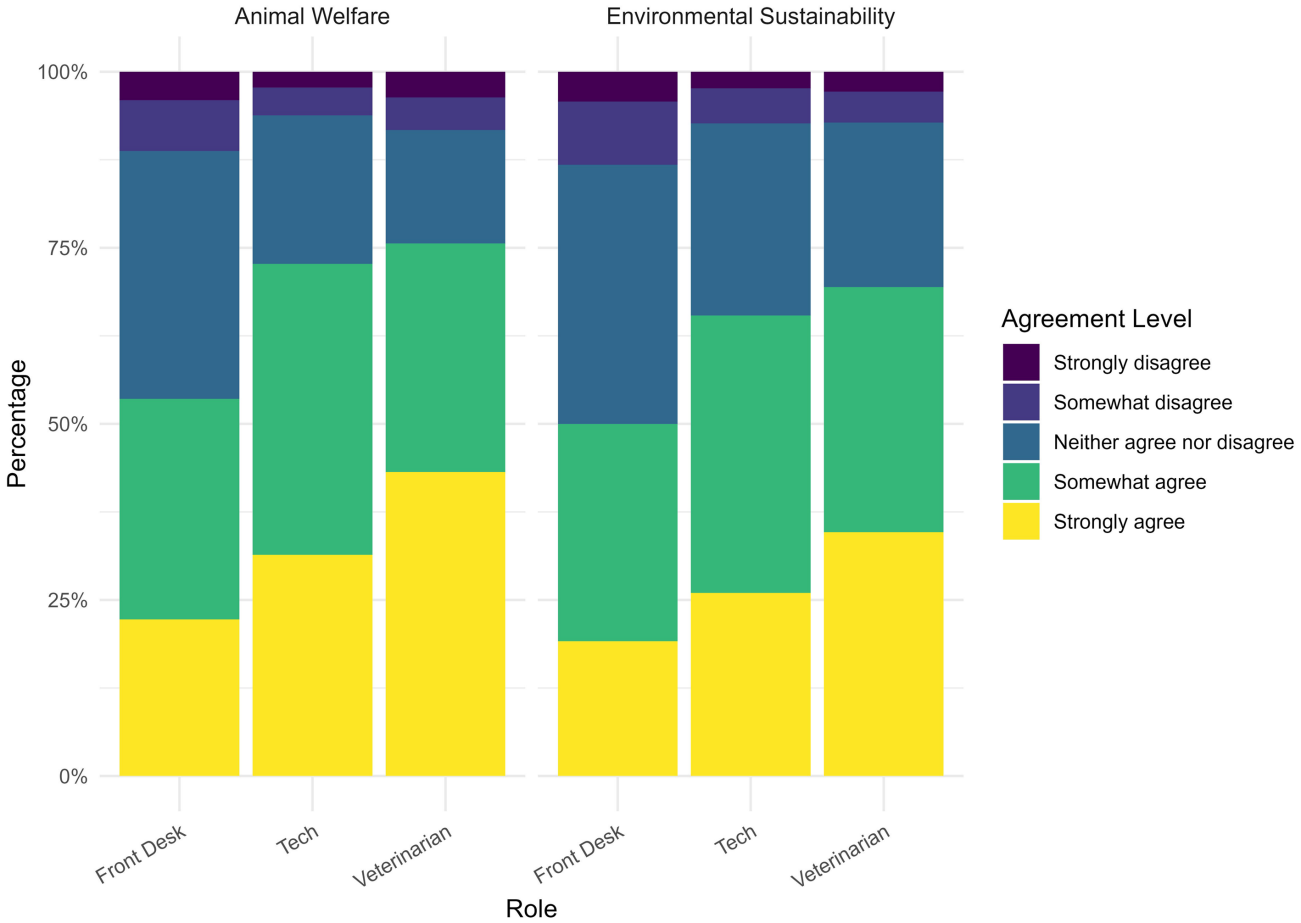
Respondent level of agreement to the statement “I trust the following sources of information regarding animal welfare standards (AW)/environmental sustainability (ES) for pet food”.

Participants assessed their trust in three positions of veterinary staff as a source of information: veterinarians, veterinary technicians, and front desk staff (Figure 4). At least 50% of participants somewhat or strongly agreed that they trusted all levels of veterinary staff to discuss either topic. For both AW and ES, the trust was significantly higher for veterinarians than veterinary technicians (OLR, AW = 0.3248716, *p <* 0.001, ES = 0.2737885, *p <* 0.001) and the front desk staff (OLR, AW = 0.9627887, *p <* 0.001, ES = 0.8199247, *p <* 0.001). Additionally, trust was significantly higher for veterinary technicians than the front desk (OLR, AW = 0.6379039, *p <* 0.001, ES = −0.5461051, *p <* 0.001). Trust was highest for veterinarians for both AW (75.7%) and ES (69.4%). Trust was lowest for the front desk staff for AW (53.5%) and ES (50%). Trust was significantly higher for veterinarians (OLR, v = 0.3091135, *p <* 0.001), veterinary technicians (OLR, v = −0.2957288, *p <* 0.001), and front desk staff (OLR, v = 0.1605028, *p <* 0.05) for AW compared to ES.

**Figure 5:**
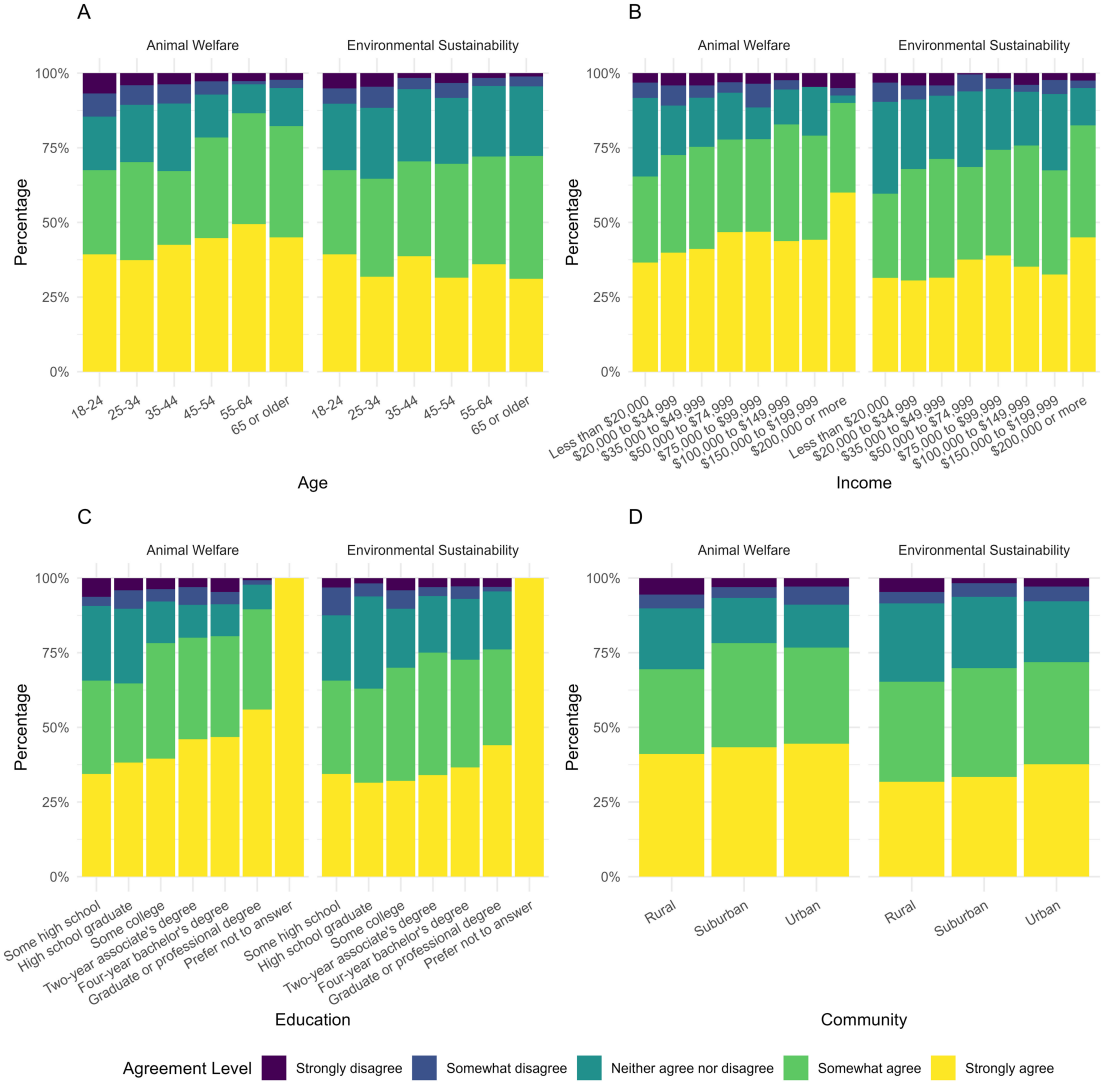
Respondent level of agreement with the veterinarian being a trusted sources of information regarding animal welfare (AW) and environmental sustainability (ES) in pet food categorized by **(A)** age, **(B)** income, **(C)** education, and **(D)** community.

We further analyzed trust in veterinarians by demographic groups (Figure 5). Increased education and income positively correlated with trust for both AW (ρs, education: correlation = 0.1601, *p <* 0.0001, income: correlation = 0.1072, *p <* 0.001) and ES (ρs, education: correlation = 0.0952, *p <* 0.05, income: correlation = 0.0896, *p <* 0.05). Increased age positively correlated with trust for AW (ρs, correlation = 0.1059, *p <* 0.001) but was not significant for ES. Community type did not significantly influence trust for either topic.

## 4 Discussion

Our survey respondents reported both ES and AW as very or extremely important factors in their pet food purchasing decisions. These findings are consistent with existing literature on the relevance of these values in driving consumer behavior (10, 12, 13). When directly compared, AW was significantly more important than ES in influencing pet food choices. This suggests that AW may be a more effective framing for brief or general conversations, as it aligns with an identified value of pet owners. However, we also identified several demographic patterns indicating that sustainability messaging may need to be refined for specific audiences.

The perceived importance of AW did not vary significantly across demographics, suggesting that it resonates broadly and could serve as a universal entry point for introducing sustainability-related topics such as sustainable procurement, third-party certifications, and the environmental co-benefits associated with improved animal health and welfare. In contrast, the importance placed on ES varied significantly by age, education, and community. Individuals with lower levels of education and younger ages were more likely to rank ES as important. Additionally, urban residents were significantly more likely to rate ES as important compared to suburban or rural areas. These patterns mirror broader consumer trends in which younger individuals tend to place greater emphasis on environmentally conscious purchasing decisions (11, 13). Given these differences, veterinary teams may consider tailoring communication strategies to reflect the values of their client base. Emphasizing AW as an area of broad agreement can serve all demographics, while bridging AW’s connection to ES may further engage specific demographic groups more effectively, including those who may not view ES as important on its own.

Additionally, AW-conscious pet food options appear to face fewer overall barriers to purchase. Respondents were significantly more likely to select cost, pet preference, and medical needs as barriers to purchasing ES-friendly pet food compared to AW-conscious options. This disparity may reflect a perception that AW-conscious products are of higher quality or healthier (20). While this is the perception, it will be important to assess nutrition separately from animal welfare criteria to ensure the pets’ nutritional needs are met, particularly when it comes to prescription diets. Not all veterinarians feel well prepared to engage in detailed nutrition discussions, and barriers to routine nutrition counseling in small animal practice have been well documented (21). These differences further support that AW may be a more effective framing for driving behavioral change, potentially leading to broader acceptance of sustainability initiatives. Cost was the most selected barrier to purchasing both ES and AW-conscious pet food. However, previous studies have found that consumers are often willing to pay more for AW-conscious pet food (7) and ES food for personal consumption (22), suggesting that cost may not be an absolute deterrent.

Third-party certifications provide transparent, independent verification of standards, helping consumers make informed choices that align with their ethical values. Third-party certifications exist for animal welfare (23), environmental sustainability (24), and a combination of both (25, 26). Most of our respondents were unaware of third-party certifications related to AW or ES, although it was unclear if they were unaware of these certifications used in the pet food industry or more broadly. Of those familiar with such certifications, most reported using them in their purchasing decisions. Among those unfamiliar with third-party certifications, the majority of participants showed interest in their use. Thus, pet food companies could benefit from seeking third-party certification and clearly displaying them on packaging to match consumer preference.

Given their overall positive perception, third-party certifications appear as a promising tool for promoting more sustainable and welfare-conscious feeding behavior going forward. They simplify decision-making by offering clear, objective criteria and can serve as effective entry points for conversations about ES and AW. With over 80% of Americans believing that corporations across economic sectors are not doing enough to address environmental issues (11), and with greenwashing decreasing consumer confidence in environmentally friendly products (27), certifications could provide credibility (28). While there are several animal welfare and environmental certification programs that could be used for pet food products or pet food companies (e.g., Certified Humane, Animal Welfare Approved, USDA Organic, or B Corporation), it is important to note that these do not assess the nutritional characteristics of the food itself.

In addition to trust in third-party certifications, most respondents agreed that veterinary staff at all levels are trusted sources on AW and ES in pet food. This indicates that the entire team is positioned to contribute meaningfully to these conversations in the clinic setting. While veterinarians were the most trusted sources, the responsibility for sustainability communication should not fall on them alone. Promoting trust in the veterinary health care team, not just the veterinarian, could help distribute this responsibility and increase the effectiveness of these conversations. Strategies to achieve this may include additional staff training, informational resources, or communication toolkits that empower all team members to confidently engage with clients on the topic of AW and ES in pet food. Veterinary staff have shown interest in sustainability in the veterinary field (29), suggesting that there is interest in further training on the subject and integrating these conversations into the veterinary clinic.

For veterinarians specifically, trust varies by demographic factors. Higher education and income were both positively correlated with trust for both AW and ES. One possible explanation for the influence of income is that financial limitations are a known barrier to accessing veterinary care (30), which may contribute to reduced trust. In our respondent pool, community type was not significantly associated with trust for either AW or ES, suggesting that veterinary team members can play a valuable role in any location. This is particularly important given that ES was rated as less important in rural and suburban areas, yet trust in veterinarians was stable, highlighting their potential to spark greater interest in sustainability regardless of geographic context. Younger age was negatively correlated with trust for AW but not for ES. This relative decline in trust among younger generations, despite increased emphasis on AW in veterinary curriculum and training (31), is concerning and warrants further attention, as it could have long-term implications for the profession’s credibility and influence in this area. Finally, trust was significantly higher for veterinarians, veterinary technicians, and front desk staff when discussing AW compared to ES. This further supports initiating conversations around AW, rather than ES, as an effective motivator for behavior change, while fostering trust within the veterinary team.

While this study provides a preliminary investigation into pet owners’ consumer preferences related to AW and ES, there are also several limitations that highlight the need for additional research in this area. While survey data on consumer choices can provide valuable insights into preferences and attitudes, stated preferences do not always translate into actual purchasing behavior. Respondents may overstate socially desirable choices or fail to account for real-world constraints such as price, availability, or habit (32). Online surveys and purchased survey panels introduce additional concerns, including sampling bias, limited representativeness, and potential lack of engagement or attention from respondents (33). For these reasons, future research validated with actual market data is needed.

## 5 Conclusion

Our findings indicate that AW resonates broadly with pet owners and may serve as an effective entry point for promoting ethical and sustainable pet food choices, while ES may require more tailored messaging. Third-party certifications can enhance consumer confidence; however, ensuring that nutritional adequacy and food safety standards are met will be critical. While additional research is needed to validate survey-stated preferences, veterinary teams appear well-positioned to encourage more sustainable and welfare-conscious feeding practices, supporting the ultimate goal of One Health: the integrated well-being of people, animals, and the environment.

## Data Availability

The raw data supporting the conclusions of this article will be made available by the authors, without undue reservation.

## Footnotes

1
https://www.centiment.co
