# Correction: Veterinary clients value animal welfare and environmental sustainability in pet food choices

**DOI:** 10.3389/fvets.2026.1818672

**Published:** 2026-04-07

**Authors:** Madison Clark, Danielle Scott, Caroline Kern-Allely, Aurora Koren, Kapahi Kawai Puaa, Jeremy Delcambre, Colleen Duncan

**Affiliations:** 1 College of Veterinary Medicine and Biological Sciences, Colorado State University, Fort Collins, CO, United States; 2 Colorado School of Public Health, Fort Collins, CO, United States; 3 School of Veterinary Medicine, Louisiana State University, Baton Rouge, LA, United States

**Keywords:** animal welfare, environmental sustainability, pet food, veterinary, veterinary team

In the published article, there was a mistake in the **Introduction**.

The second sentence of the second paragraph was displayed as “…which is roughly comparable to powering 7.5–20 billion homes for one year.” The correct statement is “…which is roughly comparable to powering 7.5–20 million homes for 1 year.”

A correction has been made to the section 1, paragraph 2:

“The ways we care for companion animals have meaningful impacts on environmental health. Pet food, for example, contributes 56–151 million Tonnes (Mt) CO_2_ equivalent (eq) annually (4), which is roughly comparable to powering 7.5–20 million homes for 1 year. In the US, where many households own pets, food consumption by dogs and cats accounts for 25–30% of the environmental impacts from the animal production industry, in terms of the use of land, water, fossil fuel, phosphate, and biocides (5). Importantly, many of the same supply chains that drive environmental impacts in pet food production also raise animal welfare (AW) concerns. Surveyed pet owners report significantly greater concern for the welfare of animals in intensive production systems than non-pet owners (6) and demonstrate a willingness to pay more for animal welfare labeled products (7). As the environmental impacts of pet food largely depends on the type and composition of these foods (8, 9) choices that owners make regarding what to feed their pets can be very impactful.”

Author Aurora Koren was erroneously spelled as Aurora Kohen.

The original version of this article has been updated.

