# Supplementary material for: Veterinary clients value animal welfare and environmental sustainability in pet food choices
Source: Front Vet Sci. 2026 Feb 10;13:1735331. doi: 10.3389/fvets.2026.1735331 (PMC12929148; doi:10.3389/fvets.2026.1735331)
Supplement: Supplementary file 1 [file Table_1.docx]

**Screening**

What pets do you take to the veterinarian?

- Dog(s)
- Cat(s)
- Dog(s) and cat(s)
- I don’t use veterinary services

**Demographics**

1. How old are you?

- 18-24
- 25-34
- 35-44
- 45-54
- 55-64
- 65 or older
- Prefer not to answer

1. What is the highest level of education you have completed?

- Some high school
- High school graduate
- Some college
- Two-year associate's degree
- Four-year bachelor's degree
- Graduate or professional degree
- Prefer not to answer

1. What is your annual household income before taxes?

- Less than $20,000
- $20,000 to $34,999
- $35,000 to $49,999
- $50,000 to $74,999
- $75,000 to $99,999
- $100,000 to $149,999
- $150,000 to $199,999
- $200,000 or more
- Prefer not to answer

1. How would you describe the community where you live?

- Urban
- Suburban
- Rural

1. What state do you live in? (drop down)

**Block 1: Importance**

1.1 How important are the following factors when making food choices for your pet?

**Environmental sustainability-** responsible management of natural resources to fulfill current needs without compromising the ability of future generations.

**Animal Welfare-** the physical and mental state of animals used for the production of meat, dairy, and eggs (disease prevention, good living conditions, good overall health, ability to express normal behaviors, comfortable, etc).

| Importance | Environmentally Sustainable | Animal Welfare |
| --- | --- | --- |
| Very Important (5) |  |  |
| Important (4) |  |  |
| Moderately important (3) |  |  |
| Slightly important (2) |  |  |
| Not important (1) |  |  |

**Block 2: Barriers**

2.1 For each of the following, what factors deter you from choosing a pet food within that category (select all that apply)

| Deterrents | Environmentally Sustainable | Animal Welfare |
| --- | --- | --- |
| Cost |  |  |
| Knowledge of options |  |  |
| Availability of options |  |  |
| nutrition |  |  |
| pet preference |  |  |
| Medical needs (e.g. food allergies or prescription diets) |  |  |
| Other (fill in) |  |  |

**Block 3: Certifications**

3.1 Are you familiar with any third-party certifications that address either environmental sustainability or animal welfare in pet food production?

| Familiarity | Environmentally Sustainable | Animal Welfare |
| --- | --- | --- |
| Yes |  |  |
| No |  |  |

3.2 If yes, rate your level of agreement with the following statement:

| Strongly agree (5) | Somewhat agree (4) | Neither agree nor disagree (3) | Somewhat disagree (2) | Strongly disagree (1) |
| --- | --- | --- | --- | --- |

3.3 If no: Third-party certifications serve to verify companies' adherence to specific sustainability standards, offering credible evidence of their green practices. Third-party certifications enable consumers to make informed, sustainable choices while reducing the risk of being deceived by misleading marketing claims. Based on this information, how likely are you to utilize third party certifications in making pet food decisions going forward?

| Very Likely (3) | Somewhat likely (2) | Not likely (1) | I Don't Know (0) |
| --- | --- | --- | --- |

**Block 4: Trusted Sources**

4.1 Please indicate your level of agreement with the following statement: I trust the following sources of information regarding **animal welfare standards** for pet food

|  | Strongly Agree (5) | Agree (4) | Neutral (3) | Disagree (2) | Strongly Disagree (1) | Not used/NA (0) |
| --- | --- | --- | --- | --- | --- | --- |
| Veterinarian |  |  |  |  |  |  |
| Veterinary Technicians |  |  |  |  |  |  |
| Veterinary Front Desk Staff |  |  |  |  |  |  |
| Other (fill in) |  |  |  |  |  |  |

4.2 Please indicate your level of agreement with the following statements: I trust the following sources of information regarding **environmental sustainability** in pet food

|  | Strongly Agree (5) | Agree (4) | Neutral (3) | Disagree (2) | Strongly Disagree (1) | Not used/NA (0) |
| --- | --- | --- | --- | --- | --- | --- |
| Veterinarian |  |  |  |  |  |  |
| Veterinary Technicians |  |  |  |  |  |  |
| Veterinary Front Desk Staff |  |  |  |  |  |  |
